# Deciphering Differential Behavior of Immune Responses as the Foundation for Precision Dosing in Allergen Immunotherapy

**DOI:** 10.3390/jpm13020324

**Published:** 2023-02-13

**Authors:** Antoine Magnan, Jean-François Nicolas, Davide Caimmi, Marc Vocanson, Thierry Haddad, Luc Colas, Silvia Scurati, Laurent Mascarell, Mohamed H. Shamji

**Affiliations:** 1 INRAe UMR 0892, Hôpital Foch, Université de Versailles Saint Quentin, Paris-Saclay, 92150 Suresnes, France; 2 CIRI-International Center for Infectiology Research, INSERM U1111, Lyon1 University, Ecole Normale Supérieure de Lyon, CNRS, UMR 5308, 69007 Lyon, France; 3 Allergy Unit, Department Respiratory Medicine and Allergy, Hôpital Arnaud de Villeneuve, University Hospital of Montpellier, 34090 Montpellier, France; 4 Dermatology, Allergology and Vascular Medicine, Tenon Hospital, 75020 Paris, France; 5 Plateforme Transversale d’Allergologie, Clinique Dermatologique, CHU de Nantes, 44093 Nantes, France; 6 UMR 1064, Center for Research in Transplantation and Translational Immunology, INSERM, Nantes Université, 44093 Nantes, France; 7 Stallergenes Greer, 92160 Antony, France; 8 National Heart & Lung Institute, Imperial College London, London SW7 2AZ, UK; 9 NIHR Imperial Biomedical Research Centre, London W2 1NY, UK

**Keywords:** allergen immunotherapy, basophils, biomarkers, clinical response, determinants, dose adaptation, immune response, innate lymphoid cells, precision dosing, T cells

## Abstract

Like in many fields of medicine, the concept of precision dosing has re-emerged in routine practice in allergology. Only one retrospective study on French physicians’ practice has addressed this topic so far and generated preliminary data supporting dose adaptation, mainly based on experience, patient profile understanding and response to treatment. Both intrinsic and extrinsic factors shape the individual immune system response to allergen immunotherapy (AIT). Herein, we focus on key immune cells (i.e., dendritic cells, innate lymphoid cells, B and T cells, basophils and mast cells) involved in allergic disease and its resolution to further understand the effect of AIT on the phenotype, frequency or polarization of these cells. We strive to discriminate differences in immune responses between responders and non-responders to AIT, and discuss the eligibility of a non/low-responder subset for dose adaptation. A differential behavior in immune cells is clearly observed in responders, highlighting the importance of conducting clinical trials with large cohorts of well-characterized subjects to decipher the immune mechanism of AIT. We conclude that there is a need for designing new clinical and mechanistic studies to support the scientific rationale of dose adaptation in the interest of patients who do not properly respond to AIT.

## 1. Introduction

The concept of precision dosing (also known as personalized or individualized dosing), which focuses on tailoring drug therapy to the needs of an individual, or group, informed by pertinent intrinsic, lifestyle, and environmental factors [1], is currently experiencing a great resurgence of interest in all fields of medicine, and particularly in allergology. At present, allergen immunotherapy (AIT) is the only available treatment option for allergic disorders that targets the underlying pathophysiology with a potential disease-modifying effect [2,3]. The clinical efficacy of AIT for respiratory allergy has been documented in randomized controlled trials for a number of allergen extracts, with the dose-response relationship assessed in phase II-III studies to obtain the best benefit–risk ratio across a patient population [4]. However, those results are obtained in a highly selected patient population, enrolled using well-defined inclusion and exclusion criteria. Moreover, adherence to treatment is also strictly followed. The spectrum of patients seen by physicians in real-life clinical settings is wider and goes beyond the ones included into randomized trials in terms of sensitization profile, clinical phenotype, concomitant health conditions or lifestyles. As an example, only 5.4% of asthmatic patients referring to specialists or general practitioners met the inclusion criteria of efficacy randomized controlled trials [5]. In addition, adherence to treatment is also often suboptimal in real-life, with an impact on treatment outcomes. Differences in clinical outcomes of patients are therefore expected between randomized and real-life studies. 

As preliminary elements, some differences were confirmed in a retrospective observational study involving allergy physicians in France [6]. In their clinical practice, nearly 80% of physicians reported adapting the dose of AIT downwards or upwards (from rarely to often) in 20% of their patients at the end of the titration phase. Downward adjustment was more frequent than upward adjustment, considering the majority of patients had been treated for 2 to 3 years. During the maintenance phase, about 95% of physicians used this dose adaptation practice in up to 19% of their patients. Dose adjustment was often upward, sustained and more frequently performed during the second year and until the end of treatment. The decision to adjust the dose was influenced by factors related to the occurrence of adverse events in 90% of the cases, efficacy of the treatment in 60% of the cases, patient sensitivity in 42% of the cases and the patient profile, including severity of symptoms, in 30% of the cases. Hence, a dose adaptation of AIT to enable patients to benefit from treatment, whilst maximizing safety and tolerability, appears to be common in real-life practice. Selecting the most appropriate dose, rather than the registered standard dose, for some patients, thus, seems crucial to tailor treatments to individual needs. Nevertheless, this dose adjustment is essentially empirical and there is a lack of scientific evidence on the relevance of dose adaptation from an immunological standpoint, and no biomarker has been identified so far for dose optimization.

The aim of this review is to identify factors explaining interpatient variability in clinical responses to AIT in conjunction with immunological responses, and to underpin the potential importance of dose adaptation in treatment modalities. As a benchmark, perspectives on dose adaptation requirements in chronic inflammatory diseases, food allergy and allergic asthma are discussed at first. Genetic and environmental factors possibly accounting for observed variations in both disease severity and interpatient response variability to AIT are subsequently addressed. Finally, this review discusses hypotheses of the dose–effect relationship between allergen concentration of therapy allergen extracts and responses of immune cell components involved in AIT mechanisms, while providing first perspectives on future research to characterize patient variability for the development of personalized treatments.

## 2. Examples of Dose Adaptation in Inflammatory and Allergic Drugs

Choosing the most appropriate treatment according to the endo-phenotype of an individual or a small group of patients is one of the key issues in precision medicine. Over the past 20 years, there has been an evolution in oncology towards precision dosing approaches [1], but with little impact in other therapeutic areas. However, some existing treatments, such as methotrexate, oral immunotherapy and immunomodulatory biologics, require dosage adaptations depending on patient-specific clinical and biological parameters.

### 2.1. Dosage Adaptation of Methotrexate in Psoriasis

Psoriasis is a multi-factorial disease, in which the onset of eruptions and worsening of symptoms are the result of genetic predisposition, skin renewal, adaptative immune system dysfunction and factors related to the patient’s environment (e.g., skin irritation, fatigue, stress, sun exposure, drugs or chronic viral infection, such as human immunodeficiency virus (HIV) infection [7]). Due to its anti-proliferative cellular effect, methotrexate (MTX) is used to treat chronic skin inflammation present in psoriasis [8]. This treatment is prescribed as either tablets or a subcutaneous injection, to be taken or performed once a week. An MTX dose varies between 7.5 and 25 mg per week (15 mg per week on average). At low doses administered for immune-mediated chronic inflammatory diseases such as psoriasis, MTX is rapidly absorbed, then eliminated unmetabolized via renal excretion and reabsorption within the renal tubules with interindividual variations [9]. It is generally accepted that the MTX dosing regimen should be adjusted to patients’ clinical response and tolerance [10]. Depending on individual disease severity and patient tolerance, the initial dose may be increased progressively by 2.5 mg per week. Doses above 20 mg per week may be associated with a significant increase in toxicity, including bone marrow suppression. Thus, after the targeted therapeutic result has been achieved, the dosage should be gradually reduced to the lowest possible effective maintenance dose. It has also been reported that MTX hematotoxicity and pulmonary toxicity may vary according to the patient age [11,12]. Hence, the use of low-dose MTX as a first-line systemic therapy for psoriasis, in consideration of interindividual variability, is a first example of dose adaptation according to the clinical profile of patients.

### 2.2. Incremental Posology in Oral Immunotherapy for Food Allergy

Immunoglobulin(Ig)E-mediated food allergies are another example of highly heterogeneous diseases [13]. For the same allergen, eliciting doses are at a variance between allergic patients [14,15]. For that reason, oral immunotherapy (OIT) protocols may vary from patient to patient. The approach to food allergy immunotherapy is analogous to that of desensitization protocols, with continued exposure to an allergen achieved through an initial dose escalation, followed by a build-up and maintenance phase. Each dose escalation is increased by approximately 25% to 100% of the preceding dose and is administered under clinical observation, before continuing the new dose at the patient’s home [16]. In some patients, dose-related adverse effects may require holding or decreasing the dosage and, in some cases, cessation of therapy. Different dosages during the maintenance phase have been tested for peanut OIT, mainly ranging from 300 to 4000 mg of protein [17]. The latter also showed that modifications of the up-dosing regimen occur in a small proportion of patients requiring dose reduction or staying at the same dose level to avoid adverse reactions. Overall, the choice of maintenance dose is a balance between achieving clinically relevant efficacy and not increasing the dose to levels resulting in an inacceptable frequency of adverse events.

### 2.3. Adaptation of Treatment Modalities for Treating Airway Allergy and Asthma Symptoms

Individualized dosing adaptations are already being performed in clinical practice for drugs indicated for patients suffering from allergic rhinitis or asthma. Examples are corticosteroids, for which a meta-analysis reports considerable individual variability in response to daily doses of inhaled corticosteroids in asthma [18], or omalizumab, a humanized anti-IgE monoclonal antibody indicated for patients with moderate to severe persistent asthma, who have a positive skin test or in vitro reactivity to a perennial aeroallergen and whose symptoms are inadequately controlled with inhaled corticosteroids. The dose and dosing frequency of omalizumab can be determined by taking into consideration serum baseline levels of total IgE (IU/mL) and the patient’s body weight (kg). Subcutaneous doses administered range between 75 mg and 600 mg in 1 to 4 injections, every 2 to 4 weeks. Total IgE levels are elevated during treatment and remain elevated for up to one year after the discontinuation of treatment [19]. Therefore, dose adjustments cannot be based on the evolution of total IgEs during therapy and can only be performed in the case of significant changes in body weight. Analyses of population pharmacokinetics data suggest that demographic characteristics, such as age, race/ethnicity, gender or the body mass index are not relevant to drive dose adjustment decisions [20].

## 3. Dose Adaptation in Respiratory Sublingual Immunotherapy

### 3.1. Intrinsic and Extrinsic Factors Leading to Individual Inter-Variability of the Immune Response to Sublingual Immunotherapy

The sources of variability in human responses to pharmacological interventions, including AIT and more specifically sublingual immunotherapy (SLIT), are starting to be elucidated and can arise from both intrinsic (i.e., genetic predispositions, gender and age) and extrinsic (i.e., environment, encompassing infections and conditions of exposure upon administration, as well as microbiota composition) factors. In the following subsections, we focus on the increasing evidence that these factors eventually shape a patient’s immunity in conjunction to variability in response to SLIT, both in terms of efficacy and tolerability.

#### 3.1.1. Intrinsic Characteristics

- Genetics and gender

Atopic diseases have a strong hereditary component, highlighted by family aggregation studies [21]. More than 30 genes have been associated with asthma or atopy-related phenotypes in genome-wide association studies. Genes for allergic disorders may be categorized into four main groups based on their major functions [22]: (1) barrier layer and defense; (2) antigen recognition and presentation; (3) immunoregulation and T helper(T_H_)2 differentiation; (4) effector targets. Allergic airway diseases are therefore highly heterogenous diseases with patient-dependent specificities. Disease severity and how a patient’s immune system will respond to the administered dose of SLIT might be partly driven by genetic predisposition and gender.

It is generally accepted that genetics of respiratory allergic diseases are multifactorial and depend on multiple genes and genetic variants, including genes regulating T_H_1 and T_H_2 differentiation and IgE production, genes modulating innate immunity and the inflammation process, or genes improving T-cell adhesion. Likewise, it could be hypothesized that variances across individuals in response to AIT partly stem from several gene polymorphisms. However, deciphering the genetic determinants of a patient’s response to AIT requires strong evidence from both independently replicated studies for association and linkage, and related functional changes. Replicated population studies and functional genomics investigation remaining uncertain, genetic studies will be challenging and costly to undertake for pharmacogenetics to be used in routine clinical practice. 

The development of high-throughput technologies, low-cost gene chip and next-generation DNA sequencing could help handle and analyze vast amounts of patient genetic data in the coming years. Functional studies and system biology approaches will undoubtedly be necessary to fully grasp allergic patient phenotypic heterogeneities, underlying endotypes and AIT molecular mechanisms, and eventually unleash personalized prescription of AIT.

Sex-dependent differences in reactivity towards AIT probably depend on several mechanisms involving both genetic and hormonal factors. In fact, gender is a clinical marker that can be used to capture the significant heterogeneity in patient response to certain treatments. 

Differential patterns of cytokine production by gender were seen in adults, with higher IL-13 and IL-10, and lower IL-5 in males, for example [23]. Alternatively, specific IgG_4_ levels after house dust mite (HDM) AIT were not significantly different between males and females at any time points [24]. With regards to hormones, a study in asthma animal models has shown that estrogen increases, and testosterone decreases T_H_2-mediated inflammation, and that sex hormones affect cytokine expression and antigen presentation by dendritic cells (DCs), as well as mast cell degranulation [25]. Premenstrual status was listed as a potential SLIT-specific risk factor for anaphylaxis, requiring investigation in prospective clinical studies [26]. Depending on these risk factors, dose reduction or treatment interruption may be required for patients. Hormones are, thus, expected to have a significant role in explaining immune differences between patients, but this role has not yet been fully explored and could be further investigated to understand gender specificities.

- Age

The age of a patient may have an impact on the patient’s response to AIT at three levels: altered immune response to the administered dose, adherence to treatment and presence of co-morbidities. Patients’ immune mechanisms evolve with age, showing similarities at both ends of the age spectrum, as neonates and elderly individuals have elevated susceptibility to various pathogens relative to adults. Neonates’ immune system is skewed toward mounting tolerogenic responses essential for tissue homeostasis, as an encounter of a barrage of new pathogens requires broad and rapid immune protection [27], while the immune system weakens in elderly individuals, partly due to atrophy of the thymus [28], defect in T-cell priming, cellular senescence or exhaustion. As an example of immune dysregulation in elderly individuals, DCs secrete enhanced levels of pro-inflammatory cytokines, which are not regulated, as the secretion of anti-inflammatory cytokine IL-10 is impaired [29]. Interestingly, some studies highlighted that elderly patients successfully respond to grass or HDM AIT for allergic rhinitis [30,31,32,33], and that a long-term effect of SLIT to either grass pollen or HDM was also observed in these patients. In addition, specific IgG_4_ levels were significantly increased after AIT, similarly to what is observed in younger individuals. A limitation of these studies is the small number of patients enrolled and the lack of functional assays to assess the blocking activities of antibodies in the sera. We cannot exclude that the production of blocking allergen-specific IgG antibodies is in turn altered for some patients. Further trials are needed to confirm these preliminary results, along with state-of-the-art mechanistic studies, including the frail elderly, to further understand how they can benefit from AIT dose adaptation. While waiting for this, elderly patients are likely to require careful individualized assessment of AIT risk/benefit ratio, and hence partly dosing regimens, considering the more frequent occurrence of co-morbidities and the consequent need of a daily-based multidrug regimen.

#### 3.1.2. Extrinsic Characteristics

- Infections

It is well established that allergic airway diseases are associated with an increased risk in infections [34]. In asthma, rhinoviruses are the major cause of asthma exacerbations, and the deficient production of interferon (IFN)-λ by rhinovirus in asthmatic primary bronchial epithelial cells and alveolar macrophages is highly correlated with clinical illness severity [35]. Although little is known about the potential influence of respiratory viral infections on AIT, oropharyngeal infections have been listed as potential SLIT-specific risk factors for anaphylaxis [26]. Viruses may interfere with the patient’s immune mechanisms. Hence, HIV infection has been regarded to be a relative contraindication for AIT. HIV invades various immune cells (e.g., CD4+ T cells and monocytes), resulting in a decline in CD4+ T cell numbers below a critical level, and the loss of cell-mediated immunity. Becoming progressively more susceptible to infections, individuals may have unpredictable responses to AIT. Therefore, for patients with acquired immunodeficiencies, AIT should be performed on an individual basis, depending on the patient’s conditions [36]. Concomitant cytomegalovirus infection may also worsen allergic symptoms through the activation of peculiar conventional DCs located in the airway mucosa, enhancing the allergenic potential of otherwise poorly allergenic environmental protein antigens [37]. Lastly, viral proteins may interfere with major histocompatibility complex (MHC)-II antigen processing and presentation pathway [38]. Altogether, this host–pathogen interplay is still not yet fully understood and implications on AIT would require additional studies, with the aim to properly address the need of individuals suffering from diverse viral infections.

- Conditions of exposure upon AIT administration

Patient’s environmental exposures (i.e., to pollen and air pollutants, with which interactions with the human body may interfere with a patient’s immune system) are likely to impact AIT outcomes. In-season dosage adjustment for pollen subcutaneous immunotherapy (SCIT) remains controversial. Some studies showed that systemic reactions (SRs) did not appear to be associated with days of peak pollen counts during mountain cedar pollen seasons, while others found that lowering doses during pollen seasons in very sensitized patients, with high positive skin test results, reduced SRs of all severity grades [39,40]. By contrast, SRs are extremely rare with SLIT, rendering the consequent need of in-season dosage adjustment less likely to occur. Air pollutants can induce oxidative stress and promote epigenetic modifications with increased gene promoter methylation, leading to immune dysregulation [41]. In asthma, ambient air pollution exposure may worsen symptoms, at least in part, by an epigenetic decrease in FOXP3 expression and impaired T regulatory (Treg) cell-mediated suppression of T_H_2 responses [42]. The impact of dose adaptation for AIT has not been tested so far in asthma situations, but it certainly highlights the importance of better phenotyping of patients undergoing AIT.

- Microbiota composition

Microbiota plays an essential role in the homeostasis and regulation of the immune system, and is consequently likely to affect the reactivity of patients to AIT due to the immunomodulating effects of intestinal microbiota. Many factors imbalance microbiota, such as antibiotic uptake [43], infections [44], stress [45] or the diet [46]. The intestinal microbiota is mainly composed of four bacterial phyla: Actinobacteria, Bacteroidetes, Firmicutes and Proteobacteria. Bacteroidetes, Firmicutes and Proteobacteria are also prevalent in the lung, and Actinobacteria and Firmicutes in the nose microbiota [47]. Since the gut microbiota impacts the function of T cells and other immune cell subsets, both within the gut-associated lymphoid tissue and beyond, microbiota-derived signals have been shown to have broad roles in the regulation of several diseases and in their treatments, and possibly in the response to AIT [48]. Moreover, microbiota-derived short-chain fatty acids (SCFAs), such as acetate, propionate and butyrate, are important energy sources for colonic cells and bacterial communities, and strengthen the gut barrier integrity by regulating the proteins involved in epithelial tight junctions and ensuring appropriate gut permeability [49]. In cancer immunotherapy, major advances have been made in the past years to understand the contribution of microbiota composition to set up successful treatment [50]. As an example, a study highlighted that gut microbiota enriched with *Faecalibacterium* and other Firmicutes is associated with a beneficial clinical response to ipilimumab in metastatic melanoma patients [51].

It can be hypothesized that bacterial strains can also modulate the effects of AIT. Specifically, activities of immune cells, including DCs, mast cells, ILCs, Breg cells and Treg cells involved in AIT mechanisms, are modulated via epigenetic modifications by microbiota metabolites. For instance, probiotic (*Lactobacillus rhamnosus* GG) or vitamin D supplementation can enhance both the clinical and immunological effects of grass SLIT in the treatment of allergic rhinitis in children, via modulation of the cytokine microenvironment and higher CD4+CD25+Foxp3+ induction. Of note is an increase in Foxp3 cell induction, that was independently associated with a better clinical effect of SLIT in children [52]. Nevertheless, many early clinical trials of probiotics against different forms of allergies found in the literature have yielded inconsistent results [53], probably reflecting the inherent complexity of allergic disorders and significant influence of patient immune specificities. Thus, major research efforts will be essential in the future to close this current knowledge gap and to ensure personalized dosage schemes for efficient disease-modifying treatment of allergic patients.

Furthermore, there is increasing evidence that mast cell functions can be modulated by commensal, symbiotic and pathogenic microorganisms, as well [47]. Microorganisms may influence mast cells activation via direct interaction or via secreted metabolites, such as SCFAs. As an example, some Lactobacillus strains inhibited IgE-mediated mast cell degranulation and subsequent late-phase reactions involving mast cells via a TLR2-dependent mechanism, with FcεRIα downregulation [54].

Altogether, there is substantial evidence suggesting that both patient-specific characteristics and external exposome are responsible for the patient’s immune system customization. The impact on individual patients’ response to AIT has not yet been deeply studied and will require further investigation.

### 3.2. Differential Behavior of Immune Responses According to Clinical Responses during AIT

The purpose of AIT and SLIT is to introduce an appropriate quantity of the allergen to re-orientate the immune system towards a tolerogenic response with long-lasting effects, without triggering adverse effects. Immune tolerance induction mechanisms, following allergen administration, have been well-documented in both animal and human studies. The sublingual route is considered a privileged site for inducing allergen-specific tolerance due to the peculiar biology of sublingual/oral immune cells. After administration under the tongue, allergen uptake by immune innate cells occurs throughout the oral cavity. Antigen-presenting cells, in particular CD207+ Langerhans cells, present in the epithelium, and macrophages located in the lamina propria exhibit a tolerogenic phenotype (e.g., production of IL-10, TGF-β and indoleamine 2,3-dioxygenase), and are preprogrammed to direct adaptive immune responses towards tolerance via an induction of regulatory T cells producing IL-10 [55,56,57,58,59]. In addition, oral tissues contain few pro-inflammatory cells, such as mast cells and eosinophils located in the submucosal tissues, which explains the excellent safety profile of the sublingual route [26,60].

Tolerance and response thresholds for SLIT might differ from patient to patient, thus justifying the need for different daily doses during the escalation and maintenance phases. In this section, we strive to analyze differential behavior of key cellular (i.e., innate lymphoid cells (ILCs), DCs, B and Tregs, basophils and mast cells), as wells as antibody (i.e., IgE, IgG_2_ and IgG_4_) responses, according to the clinical response during AIT. 

Eosinophils are also involved in the physiopathology of allergic reaction and in its resolution following SLIT [61]. Interestingly, reduced levels of eosinophilia have been observed in the airways of animals allergic to birch pollen or house dust mite after SLIT [62,63,64]. Similarly, human studies also demonstrated a statistically significant reduced nasal eosinophilia, fractional exhaled nitric oxide (an index of eosinophilic airway inflammation) or nasal eosinophil cationic protein levels in subjects allergic to pollens (birch, cedar, cypress) or house dust mites, undergoing SLIT [65,66].

**Figure 1:**
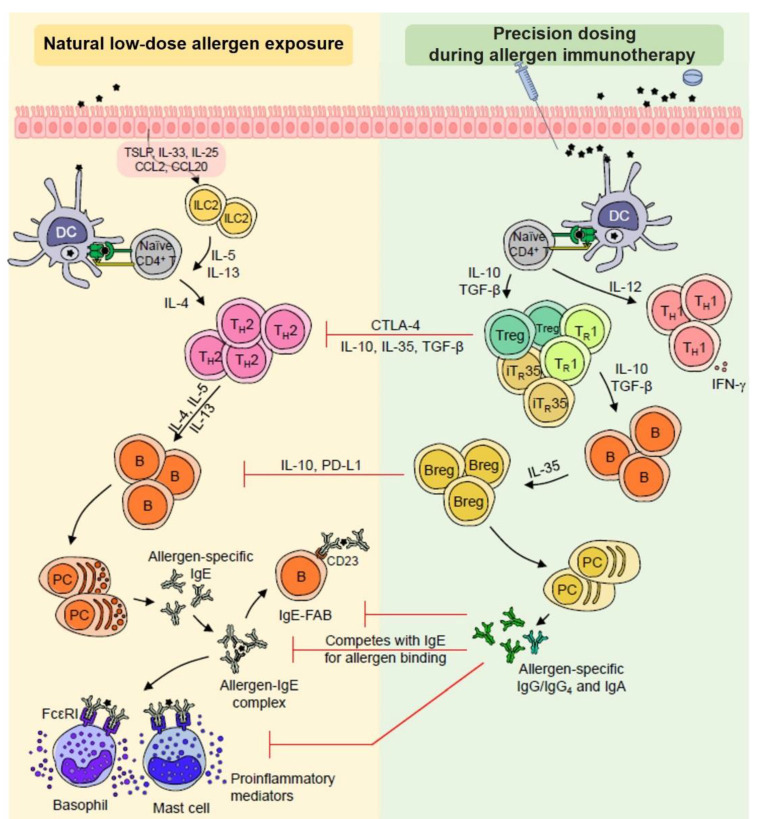
Mechanisms of allergic inflammation (in case of natural allergen exposure) and of immunological and clinical tolerance (in case of precision dosing during AIT)—Figure adapted from [3] with permission from Elsevier. Stars represent a given allergen. Breg, Regulatory B cell; DC, Dendritic cell; CCL, Chemokine ligand; CD, Cluster of differentiation; CTLA-4, Cytotoxic T-lymphocyte-associated protein 4; FcεRI, Type I Fcε receptor; IFN-γ, Interferon γ; Ig, Immunoglobulin; IgE-FAB, IgE-facilitated allergen binding; IL, Interleukin, ILC2, Group 2 innate lymphoid cell; iT_R_35, IL-35-producing Treg; PC, Plasma cell; PD-L1, Programmed death-ligand 1; TGF-β, Transforming growth factor β; T_H_, T helper; T_R_1, Type 1 Treg; Treg, Regulatory T cell; TSLP, Thymic stromal lymphopoietin.

The final aim of this analysis is to identify the most eligible individuals for dose adaptation, based on immune characteristics. Figure 1 depicted below describes mechanisms of allergic inflammation during natural allergen exposure, along with our hypothesis that a gradient of allergen concentration (dose adaptation) is needed to trigger key innate and adaptive immune cells involved in immunological tolerance, according to the patient threshold reactivity.

#### 3.2.1. Allergen Presentation Capacity of DCs and DC Polarization in the Context of AIT

DCs are a phenotypically and functionally heterogeneous leucocyte population, playing a key role in antigen/allergen uptake. Their capacity to critically activate/polarize T cells may vary from individual to individual, according to human leukocyte antigen polymorphisms or aging, for instance [67,68,69]. Currently, there are limited data on the concentrations and release time of allergens by the sublingual route for an optimal targeting of the allergen to mucosal DCs. As demonstrated by Allam et al. in an ex vivo SLIT model using human oral mucosal biopsies [57], the kinetic of grass pollen allergen, Phl p 5, uptake by oral human Langerhans cells (oLCs) is dose-dependent up to a point of saturation. Moreover, an allergen binding by oLCs enforces the production of tolerogenic cytokines (i.e., IL-10, TGF-β1) and induces TGF-β1 and IL-10-producing T cells. The latter study, thus, suggests a correlation between the allergen concentration and the strength of the tolerogenic response. From these preliminary data, we may hypothesize that there is an interest for a dose adaptation approach according to varying numbers of oLCs at a local site [70,71] as well as individual sensitivity thresholds of oLCs activation [57].

Polarization of DCs is also indicative of the patient clinical responses to AIT. Molecular markers associated with polarized DCs have been recently identified. In particular, complement component 1 (C1Q), CATC, GILZ, F13A, FKBP5, stabilin-1 (STAB1), and FcγRIII have been associated with tolerogenic regulatory DCs (DCregs), whereas CD141, GATA-3, OX40 ligand and receptor-interacting serine/threonine-protein kinase 4 (RIPK4) were associated with type 2 proallergic DCs (DC2s) [72,73], reviewed in [74]. Following a 4-month grass pollen SLIT, DC2-associated markers (i.e., CD141, GATA3, OX40 ligand and RIPK4) were downregulated concomitantly with an upregulation of DCreg-associated markers, including C1Q, FcγRIIIA, in the blood of clinical high responders, as opposed to low responders [72,73]. As such, these results suggest that molecular changes at the level of DCs could represent an early signature indicative of the subsequent orientation of adaptive immune responses during immunotherapy [72,73]. They also showed the heterogeneity of innate immune response (difference in DC polarization), in grass pollen allergic patients receiving the same therapy. The latter highlights the importance of DCs in driving the clinical response and strongly suggests that patients’ variability in DC polarization is associated with their clinical responses, which is in line with a very recent paper addressing polarization changes between LCs and DC2s in a murine epicutaneous AIT model [75]. Together, both studies analyzing allergen presentation capacity of DCs and DC polarization provide converging arguments that an optimal dose is required to achieve sufficient allergen uptake to initiate tolerogenic adaptive immune responses.

#### 3.2.2. Innate Lymphoid Cells (ILCs) in the AIT Mechanism

ILCs are a family of innate response effectors that lack antigen-specific receptors, involved in the initiation and regulation of inflammation, mainly through early cytokine secretions [2]. In intimate contact with the epithelial cells of the respiratory and intestinal mucosa, they are in the front line to respond rapidly to any disturbances in the environment [76]. ILCs main designated subtypes are ILC1, ILC2 and ILC3, with comparable cytokine profiles to T_H_1, T_H_2 and T_H_17 subsets, respectively [2]. ILC2s are mainly positioned at the barrier surfaces of the skin, airway and intestinal mucosa, and can promote inflammation in response to IL-33, IL-25 and thymic stromal lymphopoietin (TSLP). Several studies have delineated critical roles of ILC2s in allergic diseases, asthma severity and in virus-induced asthma exacerbations [2,77]. For example, increased ILC2 frequencies in the blood were seen during the grass pollen season only in patients sensitized to grass pollen allergens, but not in those unsensitized to grass pollen allergens or nonallergic control patients [78]. An increased number of the circulating CCR10+ILC2 subset was specifically found in patients with asthma, likely reflecting a higher propensity of this subset to expand before homing to the inflamed lower airways. Alternatively, IL10-producing KLRG1+ ILC2s have been identified as a regulatory ILC subset (ILC10). During AIT, both ILC2s and ILC10s are rebalanced. Grass pollen AIT was able to suppress seasonal increases of ILC2s [78,79], while ILC10s are concomitantly increased in patients’ blood and correlate with clinical symptom improvement [80], reviewed in [3]. In line with the data from grass pollen AIT, the response to HDM AIT negatively correlated with ILC2 frequencies [81], and specifically, a study demonstrated that the frequencies of ILC2s are only decreased in AIT responder patients, as opposed to non-responders [82]. The latter brings the first element, showing the crucial roles of ILC subsets in immune regulation in favor of further documenting the impact of AIT on these cells. It is also in agreement with a recent study showing that AIT induces changes in the innate immune status by reestablishing a composition and frequencies of cells (mostly ILCs), similar to those observed in healthy individuals [83]. Inter-individual variability in the ILC response to AIT may account for dose adjustment requirements to establish allergen tolerance in some patients.

#### 3.2.3. T and B Regulatory (reg) Subsets and AIT

As observed for tolerogenic innate immune responses (i.e., DCregs and ILC10s), production of IL-10 as a result of AIT has predominantly been associated with the adaptive immune compartment, particularly Treg cells [84,85,86] and Breg cells [87,88,89]. The increase in Treg cells, following AIT, is closely associated with clinical efficacy [3], concomitantly with a decrease in allergen-specific T_H_2 cells [90]. In addition, reduced methylation of the CpG site within the FOXP3 gene locus of Treg cells, thereby upregulating FOXP3 expression and suppressive function of Treg cells, has been observed following SLIT [3]. A recent study denoted that efficacy of grass pollen SLIT can be underscored by IL-35-induced regulatory T (iT_R_35) cells. Levels of IL-35 and frequencies of iT_R_35 cells were increased in SLIT patients and non-allergic individuals, when compared with that of patients with grass pollen allergy [91]. Moreover, a defect in iT_R_35 cells is associated with an increase in disease severity [92]. Regarding Breg responses, high ratios of circulating Breg/Th17, following grass pollen SCIT, correlate significantly with clinical improvement after three years [93]. In agreement with these data, another study showed that HDM SCIT results in elevated frequencies of IgA- and IgG_4_- expressing Der p 1-specific B cells, plasmablasts and IL-10+ Breg cells in AIT clinical responders only [94]. Nevertheless, such observations must also be put into the perspective of the effector cell number, function and/or magnitude of response, which may be different in patients at the start of AIT [95] and may impact its effect. Thus, according to the levels/frequencies of effector cells of each patient, different AIT dosages could be beneficial.

#### 3.2.4. The Role of Humoral Responses for Reducing Basophils and Mast Cell Threshold Sensitivity during AIT

Both mast cells and basophils expressing high-affinity receptors for the Fc fragment of IgE (FcεRI) are activated upon IgE binding, resulting in the degranulation and release of inflammatory mediators. The mode of action of AIT includes the production of varying antibody isotypes by B-cells subsequently involved in the inhibition of migration of eosinophils, basophils and mast cells to tissues, and the release of their mediators [96]. Mechanistically, SLIT induces allergen-specific IgG_1_, IgG_4_ and IgA, both locally and systematically [3]. They inhibit the formation of allergen-IgE complexes by competing with IgE for allergen binding, thereby impeding the cross-linking of FcεRI on mast cells and basophils, and hence inhibiting degranulation and histamine release. The optimal allergen concentration for basophil activation, thus, varies significantly among patients [97]. Studies highlighted suppression of basophil histamine release after AIT [98] and demonstrated that birch or grass immunotherapy-induced IgG antibodies are associated with a significant reduction in basophil allergen threshold sensitivity, with a moderate effect on basophil reactivity [99,100]. Several studies reported that the reduction in basophil allergen sensitivity after AIT is due to serological allergen blocking/binding factors, competing with the cell-bound sIgE for allergen [101]. We may postulate that in patients with high basophil and mast cell activity, higher IgG_4_ (or other blocking antibodies) levels might be needed to impede the cross-linking of allergen-IgE complexes on FcεRI on mast cells and basophils. These patients might require higher maintenance doses to induce allergen tolerance. Further studies are needed to show whether changes in basophil activity are of clinical relevance in inhalant allergen immunotherapy.

#### 3.2.5. Coordinated Immune Responses as a Novel Finding for Patient Stratification

Nevertheless, humoral surrogate markers of AIT efficacy have been investigated for decades, and serum IgE and IgG_4_ levels have been analyzed in large cohorts of patients following AIT. However, no humoral marker has been shown to correlate to clinical response [3,102]. The recent findings that grass pollen-specific serum IgG_2_, as well as frequencies of IgG_2_+ memory B cells, are up-regulated after 3 years of AIT created a new interest in allergen-specific serum IgG_2_ as a marker of AIT efficacy [103]. Moreover, a recent study assessing serum IgE, IgG_2_ and IgG_4_ levels highlighted that coordinated IgE/IgG_2_ response strongly correlated only in high versus low responders [104]. The latter highlights that a single marker is not sufficient to discriminate a clinical benefit, but that multiple markers are needed. This differential behavior in various markers of the immune response between patients is the foundation for further investigation in that direction. Other antibody subtypes, such as IgA, also raise a growing interest as discriminating markers between SCIT and SLIT. Recent data especially showed that SLIT induces higher allergen specific IgA_1/2_ levels locally and systemically than SCIT, in addition to IgG [3,105]. Whether these markers could be used as candidate biomarkers should be investigated in the future in large cohorts of well-characterized subjects.

As a conclusion of this section, we can deduce that studies of the mechanisms underlying the clinical response to AIT indicate that tolerance is induced and maintained by a complex interplay between innate, adaptive, and humoral immune responses. Recent studies highlight that coordinated humoral responses are preferably observed in high responders, but not in low or non-responders. Thus, there is an urgent interest to further decipher immune response in these patients, assuming that for a subset of them, dose adaptation either by lowering or increasing the allergen concentration may be the answer to improve AIT efficacy and/or tolerability.

## 4. Conclusions and Perspectives

In this review, we have clearly discussed that a combination of environmental, metabolic and genetic factors acts as a determinant of the patients’ immune status, which may evolve throughout the life. The consequence is that immune changes seen during AIT are at variance between individuals and that immune compartments (i.e., innate and adaptive immunity) are likely to respond differently. Main types of immune cells (i.e., DCs, ILCs, B and T cells, mast cells, basophils), as well as immune humoral markers (i.e., IgE, IgG_2_ and IgG_4_), have been analyzed in relation to AIT clinical responses. Altogether, pro-allergenic type 2 immunity is downregulated, while tolerogenic responses are increased following AIT which is particularly interesting in studies allowing patient stratifications according to their clinical responses. For subsets of individuals who experience or fear side-effects, or do not clinically respond to AIT standard posology, it is becoming increasingly obvious in the current allergology practice that dose adjustment might be an option, either by lowering or increasing the dose, both during the escalation and maintenance phase. The latter is in line with a recent study describing the daily routine of allergologists. Further prospective clinical and/or real-life studies are now needed to better assess the impact of dose adaptation on the clinical response to AIT at the patient level, using valid or accepted tools by allergologists, e.g., combined symptom and medication score and total nasal symptom score in randomized clinical trials.

New innovative research studies are further needed to assess and validate the dose–response effect on the patient’s innate and adaptive immune responses, and are thus crucial to generate evidence supporting the practice of dose adaptation of AIT. For instance, prospective clinical studies where patients could be exposed to increasing doses of a clinically relevant allergen in an allergen challenge chamber, until the threshold of symptom onset is reached, would be an interesting approach to confirm interpatient variability and continue to decipher the underlying mechanisms of dose adjustment of AIT. Building on the development of both omics approaches (e.g., metabolomics, metagenomics, transcriptomics and proteomics) and environmental monitoring technologies, these studies will unleash the discovery of biomarkers fitting patients’ response profiles. Future challenges will therefore be to link the immune mechanisms of the response to AIT to easily identifiable clinical patient profiles, while developing tools to get access to patient-specific information in a timely manner to guide drug dosing. From there, it will be possible to implement personalized approaches to allergy care to ensure optimal patient benefit.

## Data Availability

No new data were created or analyzed in this review. Data sharing is not applicable to this article.
